# The **L**ipid lowering and **O**nset of **R**enal **D**isease (LORD) Trial: A randomized double blind placebo controlled trial assessing the effect of atorvastatin on the progression of kidney disease

**DOI:** 10.1186/1471-2369-9-4

**Published:** 2008-03-18

**Authors:** Robert G Fassett, Madeleine J Ball, Iain K Robertson, Dominic P Geraghty, Jeff S Coombes

**Affiliations:** 1 Renal Research Tasmania, Launceston General Hospital, Charles St., Launceston, Tasmania, 7005, Australia; 2 School of Human Life Sciences, University of Tasmania, Launceston, Tasmania, 7250, Australia; 3 School of Human Movement Studies, University of Queensland, St. Lucia, Queensland, 4072, Australia

## Abstract

**Background:**

There is evidence that dyslipidemia is associated with chronic kidney disease (CKD). Experimental studies have established that lipids are damaging to the kidney and animal intervention studies show statins attenuate this damage. Small clinical trials, meta-analyses, observational studies and post-hoc analyses of cardiovascular intervention studies all support the concept that statins can reduce kidney damage in humans. Based on this background, a double blind randomized placebo controlled trial was designed to assess the effectiveness of atorvastatin 10 mg on slowing the progression of kidney disease in a population of patients with CKD.

**Method/Design:**

The **L**ipid lowering and **O**nset of **R**enal **D**isease (LORD) trial is a three-year, single center, multi-site, double blind, randomized, placebo controlled trial. The primary outcome measure is kidney function measured by eGFR calculated by both Modification of Diet in Renal Disease (MDRD) and Cockcroft and Gault equations. Secondary outcome measures include kidney function measured by 24-hour urine creatinine clearance and also 24-hour urinary protein excretion, markers of oxidative stress, inflammation and drug safety and tolerability.

**Discussion:**

The results of this study will help determine the effectiveness and safety of atorvastatin and establish its effects on oxidative stress and inflammation in patients with CKD.

**Trial Registration:**

ANZCTRN012605000693628

## Background

End stage kidney disease (ESKD) is a major health problem resulting in a considerable increase in morbidity and mortality, decreased quality of life, and substantial health care costs [1]. Clinical trials attempting to slow the progression of kidney disease should be a major focus of research. As treatments directed at primary kidney diseases are few, therapies have been directed towards slowing the progression of kidney disease by controlling hypertension, using angiotensin converting enzyme inhibitors (ACEI's) and angiotensin receptor blockers (ARB's) and lowering the protein intake in the diet [2-6]. Dyslipidemia has been identified as an independent risk factor for the progression of kidney disease [7]. The deleterious effect of hyperlipidemia on the progression of kidney disease is based on a number of lines of evidence.

In a large number of different animal models hyperlipidemia has been clearly shown to accelerate the progression of kidney disease [8]. There is extensive evidence for the processes involved in lipid induced kidney damage, where multiple mechanisms appear to be involved but a common initiation by hyperlipidemia is present. In addition, intervention studies have assessed the effects of statins on limiting kidney damage, again, in a variety of animal models [9,10]. In these studies statins had a beneficial effect on kidney structure and function. Not only did statins reduce proteinuria, they attenuated inflammatory processes and prevented histological changes of inflammation and fibrosis in the kidney.

A comprehensive search of the literature has found 25 randomized controlled trials (RCT's) reporting on the effects of statins on kidney function however these have been mostly of small size, short duration, with narrow inclusion and exclusion criteria, often conducted on patients with normal or stable kidney function, using a variety of statins and doses, or with poor concealment and often no placebo group. Some were also potentially affected by the inclusion of other lipid lowering agents. The results of these are best summarized in the meta-analyses described below.

The large cardiovascular clinical trials such as GREACE, CARE and TNT provide data on a significant number of subjects exposed to statins [11-13]. These studies however, were primarily designed to assess cardiovascular outcomes in subjects presenting with cardiac disease or who were at a high risk of cardiac disease. They were not specifically designed to test kidney function outcomes. Post-hoc sub-analyses are commonly performed on these large trials to extract maximum benefit from the available data that takes years to accumulate at a high cost. Wang et al reported that such analyses introduce challenges and may lead to "overstated and misleading results" [14]. The most important conclusion that can be drawn from these cardiovascular studies is that subjects with kidney dysfunction who meet the cardiovascular criteria of these trials, which for most include a prior myocardial infarction, will do well from the kidney function perspective when treated with a statin. In addition there are no data available from these cardiovascular trials regarding the kidney disease diagnosis thus no conclusions can be made regarding how to treat any specific kidney disease type.

There have been two published meta-analyses reporting on the effects of statins on kidney function, one by Fried et al [15], and the most recent, reported by Sandhu et al (2006), that includes most of the 25 RCT's mentioned above [16]. The subject numbers are substantially larger in the Sandhu analysis as the larger cardiovascular trials were included. This however is a limitation as this inclusion mixes prospective studies with post-hoc, sub-studies of major randomized controlled trials. Thus the authors' conclusions must be interpreted with caution. The meta-analyses support the suggestion that statins have a beneficial effect on kidney function. The Sandhu et al meta-analysis also implies that statins have a more protective effect on kidney function in subjects recruited with a cardiovascular disease and that the more potent statin, atorvastatin, has a greater effect on protecting kidney function than the less potent statins such as pravastatin, fluvastatin, lovastatin and simvastatin [16]. Based on the need for further clarification and investigation of the role of statins in slowing CKD progression the LORD Trial was designed. This trial is intended to have inclusion criteria that reflected common clinical practice to enable the results to be as clinically relevant as possible.

## Methods/Design

### Study Design and Setting

The study was a single centre, multi-site three-year, double blind randomized placebo controlled trial in subjects with CKD. The study was conducted in North and North West Tasmania and the principal investigator is the sole nephrologist in this region.

### Ethical Considerations

The Tasmanian Statewide Scientific Committee and the Tasmanian Statewide Ethics Committee approved the LORD Trial. The Ethics Committee will be provided with annual reports of the LORD trial progress and will promptly receive all adverse event reports.

### Identification of Eligible Patients

The principal investigator and the clinical trial coordinators will screen the medical records of patients prior to their attendance at the renal clinics. The serum creatinine is measured and the results available prior to the clinic. Eligible patients will have a copy of the Ethics Committee approved patient information sheet placed in the medical record. The principal investigator will then explain the study during the clinical consultation. After the explanation the subject will be provided with a patient information sheet and informed consent form. The subject will then be asked to take this away with them and arrangements will be made to follow up via a telephone call. If the subject agrees to participate, they will sign the consent form with an independent person signing as a witness to this process.

### Eligibility

Inclusion criteria are; age 18 – 85 years, serum creatinine > 120 μmol/l at the screening visit, history of CKD, signed informed consent. Subjects will be excluded if they are already taking lipid-lowering medication or if they are females of childbearing age, able to conceive and not using contraception. Other exclusion criteria are: subjects with acute liver disease or unexplained persistent elevations of serum transaminases, or a history of alcoholism,. Patients will also be excluded if they have had a seizure within a year of study entry, have a hypersensitivity to atorvastatin or one of its components or are participating in, or propose to participate in, another clinical investigational drug study within 30 days prior to study entry.

### Randomization

The Clinical Trial Pharmacist at the Launceston General Hospital, who is independent of the study team, will perform the randomization. Subjects will also be stratified according to three diagnostic groups; glomerulonephritis, diabetes and other diagnoses. Computer generated random numbers will be placed in blocks of 10 by the Clinical Trial Pharmacist and related to a series of drug code numbers. Each drug code assignment block will refer to one of the stratification groups. Data recorded will include the drug code number, which becomes the subjects LORD trial identification number, the patient hospital record number, the date of allocation as well as the diagnosis. The sheet containing the blocks of drug code numbers are given to the clinical trial coordinator who thus is blinded to the allocation performed by the pharmacist. Once the diagnosis is confirmed by the principal investigator the clinical trial coordinator selects the next available drug code number from the relevant stratification block according to diagnostic grouping. The drug code number is then written on the prescription form and given to the subject who then presents it to the pharmacy. This enables the pharmacist (independent of the trial pharmacist) to dispense from the appropriate randomization group. The subject receives a specific purpose numbered LORD Trial plastic container with tablets enclosed, which are indistinguishable whether they contain atorvastatin 10 mg or identical placebo. The randomization code is kept sealed in an opaque envelope in the Launceston General Hospital Pharmacy Department and an identical copy is kept at an off-site location of one of the associate investigators for the duration of the study.

### Study Medication and Dosing

Pfizer Pharmaceuticals, New York provided the atorvastatin 10 mg and identical placebo, which were shipped in containers from New York. Tablets will be checked to ensure they will not exceed their expiry date at any stage of their prescription to subjects. They will then be placed in number coded containers so it is not possible to distinguish, other than with the code, which container has placebo or atorvastatin.

### Primary Objective and Primary Outcome Measure

The primary objective of the LORD trial is to assess the effect of atorvastatin 10 mg on the rate of decline of kidney function in subjects with CKD. The primary outcome measure will be the rates of decline in eGFR (ml/min/1.73 m^2^/month) measured by MDRD and Cockcroft Gault equations.

The hypothesis is that atorvastatin 10 mg will significantly slow the rate of decline of kidney function (eGFR) in subjects with CKD.

The selection of the primary outcome measure was based on the following issues: 1) the widespread use of this marker in similar clinical trials [17-23], 2) other measures including "hard" end points such as commencement of dialysis, transplantation and death were considered however trials that have used such hard end points typically recruit patients with more advanced kidney disease and in larger numbers in order to show an effect of the therapy over 2 – 5 years [2] Furthermore, although hard end points are indicators of a drug's efficacy in reducing cardiovascular events or preserving kidney function, they do not assess the impact of a treatment on altering the natural history of earlier stages of CKD. For clinical trials of subjects with all but the most advanced renal disease, use of intermediate end points of CKD progression is the only practical option for assessment of treatment efficacy and effectiveness [24], 3) intermediate endpoints that were also considered for the major outcome measure included changes in creatinine clearance, serum creatinine, proteinuria or albuminuria. However measures of serum creatinine and creatinine clearance have been argued to be less sensitive markers of the progression of CKD [25]. Furthermore, proteinuria and albuminuria may be affected by dietary changes and are therefore problematic in a longer term (three-year) study and 4) other more accurate measures of kidney function such as ^51^CrEDTA, ^99^TcDTPA and cystatin C were considered either too expensive and/or time consuming for subjects in this study population.

### Secondary Objectives, Outcome Measures and Sub Studies

The secondary objectives (and their associated measures) will assess the effects of atorvastatin 10 mg on 24-hour urine creatinine clearance and protein excretion, the requirement for ESKD management, self reported health (SF-36 questionnaire), physical activity levels (Active Australia questionnaire) nutritional status (four-day diet diary), cardiovascular events, mortality, hospital admissions, drug safety and tolerability. In addition, further secondary objectives included assessing the effects of atorvastatin on measures of inflammation (plasma C reactive protein, tumor necrosis factor-alpha and interleukin 6) and oxidative stress (plasma isoprostanes). Sub-studies on smaller groups will also assess the effect of atorvastatin on arterial stiffness and cognitive function.

### Visit One (baseline data)

**Figure 1:**
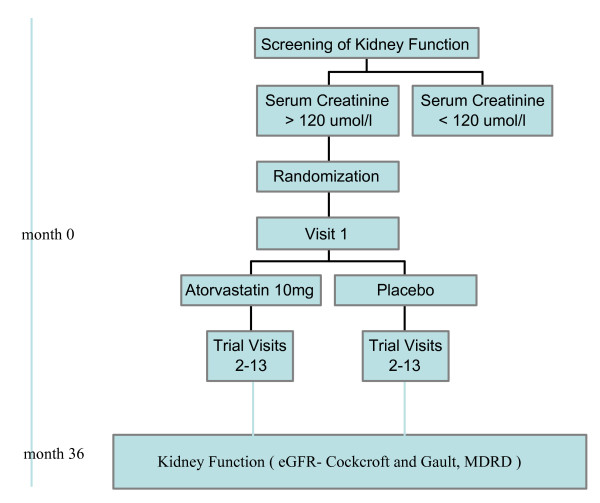
Flow chart of LORD trial.

The LORD study flow is summarized in Figure 1 and the study evaluations are outlined in table 1. After obtaining informed consent patients will be provided with a LORD Trial pathology request form, a four-day diet diary and a 24-hr urine collection bottle. They will be asked to attend the pathology laboratory at least seven days before their first trial visit (approximately three months later) to have a fasting blood sample collected and hand in a 24-hr urine sample. At the first trial visit, additional data will be obtained from the medical records (medical history, medications), measures of height, weight and blood pressure will be made and two questionnaires will be completed (SF 36 and Active Australia). At this visit they will provide their completed four-day diet diary and be given their next appointment time for trial visit two that will be approximately three months after visit one. At the completion of visit one, the clinical trial coordinator will provide patients with a prescription containing their drug code number. They then present thus to the pharmacy department to obtain their first three-month supply of trial medication as described in the section on randomization. Subjects will be instructed to take one tablet each day at any time of the day, either with or without food. The same instructions are provided on the medication container. At this visit the subjects will be given a LORD trial pathology request form and a 24-hr urine collection bottle. They will be asked to attend the pathology laboratory at least seven days prior to their next visit to have a fasting blood sample collected and hand in a 24-hr urine sample. They are given their next appointment time that will be no longer than three months after this visit. The capture of data occurs at the scheduled study visits and are transcribed onto case recoerd forms (CRFs) for entry into a specifically designed LORD Trial database.

**Table 1:** Baseline and ongoing data collection

**Baseline data and follow-up data every three months for 36 months**
eGFR Cockcroft and Gault
eGFR MDRD
24 hour urine creatinine clearance
24 hour urine protein
Weight and blood pressure
Adverse events and concomitant therapy
Laboratory tests
Hematology
Biochemistry
Oxidative stress measures
Inflammation measures
Lipid profile
**Baseline data and follow-up data at 9,18,27 and 36 months**

SF 36
Active Australia Questionnaire
Four-day diet diary

### Additional Trial Visits

These visits will occur with the principal investigator and the clinical trial coordinator every three months for a total of three years. Subjects will be required to visit the pathology laboratory and have a fasting blood sample collected and hand in a 24-hr urine collection at least 7 days before each of these visits. They are also required to return the tablet container with the remaining tablets and they will be issued with a prescription form so they can obtain a new container with a further three-month supply of trial medication from the pharmacy department. At these visits they are given a routine medical examination where blood pressure, height and weight are measured. Every nine months during this period they are given a four-day diet dairy and required to return it at the next visit. Every 9 months they are also asked to complete the SF-36 and Active Australia questionnaires. At each visit they are given their next visit appointment time.

### Adherence to Study Medication

Trial visits are conducted every three months and as a consequence, sufficient medication is included in the container to last for this interval. However as a crosscheck of the subjects adherence to drug therapy, each container has 100 tablets placed in it for each trial visit and the subjects are not informed that the containers have additional tablets. At each trial visit the subject is required to return the container. Two separate tablet counts are then conducted one by the trial coordinator and the trial pharmacist performs a separate count. This enables a check on adherence to therapy by calculating the difference between 100 and the days of therapy and comparing this with number of tablets left in the container.

### Withdrawal from Study

Subjects will be withdrawn from the study at their request, without prejudice, as documented and explained at the time of consenting. Patients who withdraw will be encouraged to consent to allow access to their pathology results for the remainder of the trial duration to enable an intention to treat data analysis.

### Sample size calculation

The following assumptions were made for the sample size calculation. The rate of decline of eGFR, based on the expected population to be recruited in the LORD trial and a similar trial [3], was assumed to be 0.29 ml/min/1.73 m^2^/month. To demonstrate a clinically significant improvement, based on the REIN study, we expected a difference in rate of eGFR decline of 40%, between atorvastatin treated subjects and controls [2]. Thus we assumed that subjects in the atorvastatin group would have a monthly decline of eGFR of 0.17 ml/min/1.73 m^2^. The standard deviation was assumed to be twice the decline (0.24) [3]. Therefore, it was estimated that the study required 63 subjects per group for alpha to equal 0.05 (two-tailed test) and a power (1-β) of 0.80. To achieve this we aimed to recruit 200 subjects into the LORD trial, which allowed for a dropout rate of 60%.

### Statistical analysis

The primary analysis will use an intention to treat approach and the secondary analysis will use an on treatment approach. The rate of decline of eGFR in ml/min/1.73 m^2^/month will be compared between atorvastatin treated subjects and the placebo group using a two-tailed independent t test. Significance will be deemed p < 0.05.

## Discussion

The objective of the LORD Trial is to assess whether atorvastatin 10 mg can slow the progression of CKD. Measures of oxidative stress and inflammation will be used to assess whether these are concomitantly affected by this statin treatment. If statins slow the progression of CKD, then most CKD patients may benefit from this treatment. Previous studies have been conducted in this area but have been either smaller, open labeled or short duration. Other studies, such as the SHARP study [26], seek to assess the same question however they include the cholesterol absorption inhibitor ezetimibe in the treatment group, which may confuse the nature of the potential effective agent. The measure of eGFR every three months during the three-year trial is another strength of this protocol. We predict atorvastatin will slow the progression of CKD.

## Competing interests

The author(s) declare that they have no competing interests.

## Authors' contributions

RGF and JSC were responsible for the design of this clinical trial, the construction of the protocol and writing this manuscript. IKR provided statistical advice. MJB and DPG provided advise and manuscript review. All authors read and approved the final manuscript.

## Pre-publication history

The pre-publication history for this paper can be accessed here:


